# Bis[4-(4-pyridyl)pyridinium] μ-4,4′-bipyridine-bis­[tetra­aqua­(4,4′-bipyridine)manganese(II)] bis­(5-sulfonatobenzene-1,3-dicarboxyl­ate) 4,4′-bipyridine solvate penta­deca­hydrate

**DOI:** 10.1107/S1600536809028359

**Published:** 2009-07-22

**Authors:** Bing-Yu Zhang, Jing-Jing Nie, Duan-Jun Xu

**Affiliations:** a Department of Chemistry, Zhejiang University, People’s Republic of China

## Abstract

The crystal structure of the title compound, (C_10_H_9_N_2_)_2_[Mn_2_(C_10_H_8_N_2_)_3_(H_2_O)_8_](C_8_H_3_O_7_S)_2_·C_10_H_8_N_2_·15H_2_O, consists of dinuclear Mn^II^ complex cations, sulfonato­benzene­dicarboxyl­ate trianions, 4-(4-pyridyl)pyridinium cations, uncoordin­ated 4,4′-bipyridine and uncoordinated water mol­ecules. One 4,4′-bipyridine mol­ecule bridges two Mn atoms, forming a centrosymmetric dinuclear complex; the mid-point of the C—C bond linking the pyridine rings of the bridging ligand is located on an inversion center. Each Mn^II^ atom is coordinated by four water and two 4,4′-bipyridine mol­ecules in a distorted octa­hedral geometry. The Mn^II^ atom deviates by 0.591 (5) and 0.209 (2) Å from the mean planes of the coordinated pyridine rings. In the 4-(4-pyridyl)pyridinium cation, the two pyridine rings are twisted with respect to each other, making dihedral angle of 34.78 (17)°. The uncoordinated bipyridine mol­ecule is also centrosymmetric. One of uncoordinated water mol­ecules has site symmetry 2, and the other uncoordinated water mol­ecule is located close to an inversion center and its one H atom is disordered equally over two sites. Extensive π–π stacking between pyridine rings is observed and an extensive hydrogen-bonding network of the types N—H⋯N, O—H⋯N and O—H⋯O is present.

## Related literature

For the nature of π-π stacking, see: Deisenhofer & Michel (1989 ▶); Xu *et al.* (2007 ▶); Li *et al.* (2005 ▶). For non-coplanar 4,4′-bipyridine or 4,4′-bipyridinium, see: Bowes *et al.* (2003 ▶); Pedireddi & PrakashaReddy (2003 ▶); Charmant *et al.* (2003 ▶); Madhu & Das (2004 ▶).
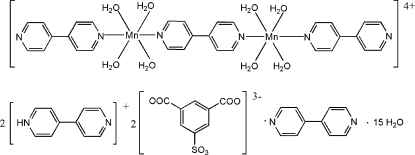


## Experimental

### 

#### Crystal data

(C_10_H_9_N_2_)_2_[Mn_2_(C_10_H_8_N_2_)_3_(H_2_O)_8_](C_8_H_3_O_7_S)_2_·C_10_H_8_N_2_·15H_2_O
*M* *_r_* = 1949.70
Monoclinic, 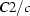

*a* = 45.393 (13) Å
*b* = 10.946 (3) Å
*c* = 19.641 (6) Å
β = 112.704 (9)°
*V* = 9003 (5) Å^3^
*Z* = 4
Mo *K*α radiation
μ = 0.42 mm^−1^
*T* = 294 K
0.30 × 0.22 × 0.20 mm

#### Data collection

Rigaku R-AXIS RAPID IP diffractometer
Absorption correction: multi-scan (*ABSCOR*; Higashi, 1995 ▶) *T*_min_ = 0.84, *T*_max_ = 0.92
50211 measured reflections
8729 independent reflections
6521 reflections with *I* > 2σ(*I*)
*R*_int_ = 0.054

#### Refinement

*R*[*F* ^2^ > 2σ(*F* ^2^)] = 0.053
*wR*(*F* ^2^) = 0.157
*S* = 1.03
8729 reflections
582 parameters
H-atom parameters constrained
Δρ_max_ = 0.41 e Å^−3^
Δρ_min_ = −0.70 e Å^−3^

### 

Data collection: *PROCESS-AUTO* (Rigaku, 1998 ▶); cell refinement: *PROCESS-AUTO*; data reduction: *CrystalStructure* (Rigaku/MSC, 2002 ▶); program(s) used to solve structure: *SIR92* (Altomare *et al.*, 1993 ▶); program(s) used to refine structure: *SHELXL97* (Sheldrick, 2008 ▶); molecular graphics: *ORTEP-3 for Windows* (Farrugia, 1997 ▶); software used to prepare material for publication: *WinGX* (Farrugia, 1999 ▶).

## Supplementary Material

Crystal structure: contains datablocks I, global. DOI: 10.1107/S1600536809028359/hk2741sup1.cif

Structure factors: contains datablocks I. DOI: 10.1107/S1600536809028359/hk2741Isup2.hkl

Additional supplementary materials:  crystallographic information; 3D view; checkCIF report

## Figures and Tables

**Table 1:** Selected bond lengths (Å)

Mn—N1	2.323 (2)
Mn—N3	2.311 (2)
Mn—O1	2.158 (2)
Mn—O2	2.156 (2)
Mn—O3	2.178 (2)
Mn—O4	2.192 (2)

**Table 2:** Hydrogen-bond geometry (Å, °)

*D*—H⋯*A*	*D*—H	H⋯*A*	*D*⋯*A*	*D*—H⋯*A*
N4—H4*N*⋯N6	0.96	1.79	2.725 (4)	163
O1—H1*C*⋯N5^i^	0.95	1.86	2.809 (4)	173
O1—H1*D*⋯O6^ii^	0.95	1.81	2.731 (3)	165
O2—H2*C*⋯O5^ii^	0.81	1.95	2.735 (3)	163
O2—H2*D*⋯O8^iii^	0.89	1.81	2.691 (3)	169
O3—H3*C*⋯N2^iv^	0.90	1.86	2.742 (4)	168
O3—H3*D*⋯O10	0.95	1.81	2.754 (3)	169
O4—H4*C*⋯O11	0.95	1.88	2.805 (4)	164
O4—H4*D*⋯O1*W*	0.87	1.85	2.706 (3)	166
O1*W*—H1*A*⋯O5	0.94	1.87	2.815 (3)	177
O1*W*—H1*B*⋯O7^v^	0.97	1.77	2.719 (3)	166
O2*W*—H2*A*⋯O4*W*^vi^	0.95	2.07	2.822 (6)	135
O2*W*—H2*B*⋯O7	0.92	1.99	2.903 (4)	175
O3*W*—H3*A*⋯O2*W*	0.95	1.79	2.694 (5)	157
O3*W*—H3*B*⋯O9	0.96	1.88	2.831 (4)	170
O4*W*—H4*A*⋯O11	0.89	2.15	2.946 (5)	148
O4*W*—H4*B*1⋯O4*W*^vii^	0.94	2.02	2.900 (8)	156
O5*W*—H5*A*⋯O8	0.94	1.92	2.772 (6)	149
O5*W*—H5*B*⋯O6*W*^viii^	0.93	1.94	2.771 (9)	147
O6*W*—H6*A*⋯O6	0.99	1.81	2.768 (6)	162
O6*W*—H6*B*⋯O7*W*^viii^	0.94	1.73	2.358 (12)	121
O7*W*—H7*A*⋯O5	0.91	2.23	3.124 (10)	166
O7*W*—H7*B*⋯O5*W*^ix^	0.90	1.76	2.291 (11)	115
O8*W*—H8*A*⋯O9	0.91	2.00	2.871 (7)	159

Symmetry codes: (i) 
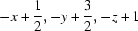
; (ii) 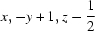; (iii) 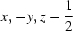; (iv) 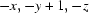; (v) 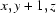; (vi) 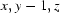; (vii) 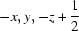; (viii) 
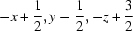
; (ix) 
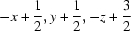
.

**Table 3:** A summary of the distances and angles between partially overlapped pyridine rings (Å, °)

Ring (*I*)	Ring (*J*)	Angle	Perp(*I*)	Perp(*J*)	*Cg*–*Cg*
N1-pyridine	N2^i^-pyridine	8.29	3.404	3.491	3.691 (2)
N2-pyridine	N6^ii^-pyridine	5.33	3.403	3.391	3.794 (2)
N3-pyridine	N5^iii^-pyridine	10.91	3.260	3.477	3.751 (2)
N5-pyridine	N5^i^-pyridine	0.00	3.544	3.544	3.547 (2)

Symmetry codes: (i) 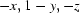; (ii) 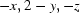; (iii) 
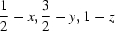
. Notes: Angle: dihedral angle between ring (*I*) and ring (*J*). Perp(*I*) is the perpendicular distance of centroid of ring (*I*) on ring (*J*). Perp(*J*) is the perpendicular distance of centroid of ring (J) on ring (*I*). *Cg*–*Cg* is the distance between centroids of ring (I) and ring (J).

**Fig. 1.**
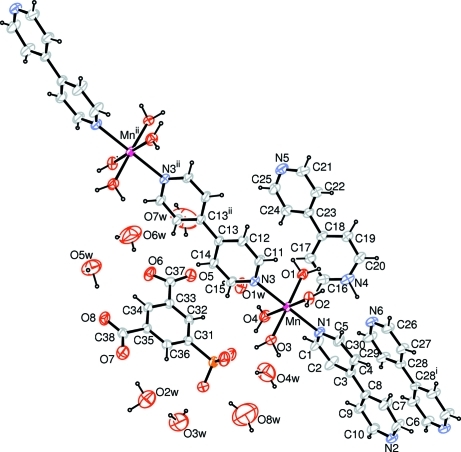
The molecular structure of the title compound with 40% probability displacement ellipsoids for non-H atoms (arbitrary spheres for H atoms) [symmetry codes: (i) -x, 2 - y, -z; (ii) 1/2 - x, 1/2 - y, 1 - z].

**Fig. 2.**
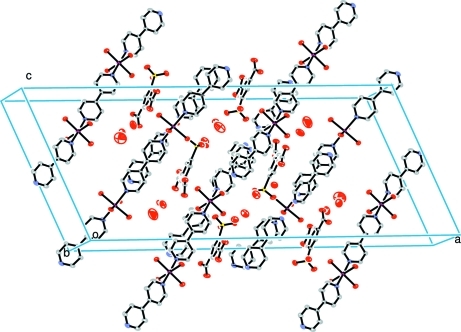
The unit cell packing diagram showing the partially overlapped arrangement between pyridine rings. H atoms have been omitted for clarity.

## supplementary crystallographic information

### Comment

The π-π stacking between aromatic rings has attracted our much attention because it is correlated with the electron transfer process in some biological systems (Deisenhofer & Michel, 1989). As a part of our ongoing investigation on the nature of π-π stacking (Xu *et al.*, 2007; Li *et al.*, 2005), the title compound incorporating 4–4'bipyridine was prepared in the laboratory and its crystal structure is reported here.

The crystal structure of the title compound consists of dinuclear Mn^II^ complex cations, sulfobenzendicarboxylate anions, 4-(4-pyridyl)pyridinium cations, uncoordinated 4,4'-bipyridine and lattice water molecules.

The dinuclear Mn^II^ complex cation is centrosymmetric. Each Mn atom is coordinated by four water and two bipyridine molecules with a distorted octahedral geometry (Table 1). The bridge bipyridine ligand links two Mn atoms to form the dinuclear complex. The mid-point of C—C bond linking pyridine rings of the bridge ligand is located on an inversion center. The Mn atom is not coplanar with the coordinated pyridine rings but deviated from the pyridine planes by -0.591 (5) and 0.209 (2) Å, respectively.

The sulfobenzendicarboxylate anion is not coordinated to the Mn atom but links with the complex *via* O—H···O hydrogen bonding (Table 1). A 4-(4-pyridyl)pyridinium cation occurs in the asymmetric unit of the crystal structure to balance the charge. In the pyridinium cation two pyridine rings are twisted to each other with a dihedral angle of 34.78 (17)°, similar to those found in the crystal structures containing 4,4'-bipyridine (Bowes *et al.*, 2003; Pedireddi & PrakashaReddy, 2003) or 4,4'-pyridinium cation (Charmant *et al.*, 2003; Madhu & Das, 2004).

In the crystal structure there are uncoordinated bipyridine and water molecules. The uncoordinated bipyridine molecule is centrosymmetric with the mid-point of C28—C28^i^ bond located in an inversion center [symmetry code: (i) -*x*, 2 - *y*, -*z*]. One of lattice water molecules has site symmetry 2, and the other lattice water molecule is located close to an inversion center and its one H atom is equally disordered over two sites.

The extensive hydrogen bonding network is present in the crystal structure (Table 2). The partially overlapped arrangement between pyridine rings is observed in the crystal structure (Fig. 2). The shorter centroid-to-centroid distances (Table 3) suggest the existence of extensive π-π stacking between pyridine rings.

### Experimental

Reagents and solvent were used as purchased without further purification. 4,4'-Bipyridine (0.16 g, 1 mmol), Na_2_CO_3_ (0.11 g, 1 mmol), sodium 1-sulfo-benzene-3,5-dicarboxylate (0.25 g, 1 mmol) and MnCl_2_.4H_2_O (0.20 g, 1 mmol) were dissolved in ethanol-water (10 ml, 1:4). The mixture was transferred into a Teflon-lined stainless steel vessel (25 ml). The autoclave was sealed and heated at 403 K for 3 d. After cooling to room temperature the mixture was filtered. Single crystals of the title compound were obtained from the filtrate after one day.

### Refinement

H atom bonded to N atom was located in a difference Fourier map and refined as riding in as-found relative position, *U*_iso_(H) = 1.5*U*_eq_(N). Water H atoms were placed in chemical sensible positions and refined in riding mode, among which the H4B was equally disordered over two sites, *U*_iso_(H) = 1.5*U*_eq_(O). Other H atoms were placed in calculated positions with C—H = 0.93 Å and refined in riding mode with *U*_iso_(H) = 1.2*U*_eq_(C).

### Crystal data

**Table:**

(C_10_H_9_N_2_)_2_[Mn_2_(C_10_H_8_N_2_)_3_(H_2_O)_8_](C_8_H_3_O_7_S)_2_·C_10_H_8_N_2_·15H_2_O	*F*(000) = 4080
*M**_r_* = 1949.70	*D*_x_ = 1.438 Mg m^−^^3^
Monoclinic, *C*2/*c*	Mo *K*α radiation, λ = 0.71073 Å
Hall symbol: -C 2yc	Cell parameters from 9036 reflections
*a* = 45.393 (13) Å	θ = 1.8–25.0°
*b* = 10.946 (3) Å	µ = 0.42 mm^−^^1^
*c* = 19.641 (6) Å	*T* = 294 K
β = 112.704 (9)°	Prism, yellow
*V* = 9003 (5) Å^3^	0.30 × 0.22 × 0.20 mm
*Z* = 4	

### Data collection

**Table:**

Rigaku R-AXIS RAPID IP diffractometer	8729 independent reflections
Radiation source: fine-focus sealed tube	6521 reflections with *I* > 2σ(*I*)
graphite	*R*_int_ = 0.054
ω scans	θ_max_ = 26.0°, θ_min_ = 1.0°
Absorption correction: multi-scan (*ABSCOR*; Higashi, 1995)	*h* = −55→54
*T*_min_ = 0.84, *T*_max_ = 0.92	*k* = −13→12
50211 measured reflections	*l* = −24→24

### Refinement

**Table:**

Refinement on *F*^2^	Primary atom site location: structure-invariant direct methods
Least-squares matrix: full	Secondary atom site location: difference Fourier map
*R*[*F*^2^ > 2σ(*F*^2^)] = 0.053	Hydrogen site location: inferred from neighbouring sites
*wR*(*F*^2^) = 0.157	H-atom parameters constrained
*S* = 1.03	*w* = 1/[σ^2^(*F*_o_^2^) + (0.0798*P*)^2^ + 12.578*P*] where *P* = (*F*_o_^2^ + 2*F*_c_^2^)/3
8729 reflections	(Δ/σ)_max_ = 0.003
582 parameters	Δρ_max_ = 0.41 e Å^−^^3^
0 restraints	Δρ_min_ = −0.70 e Å^−^^3^

### Special details

**Table:**

Geometry. All e.s.d.'s (except the e.s.d. in the dihedral angle between two l.s. planes) are estimated using the full covariance matrix. The cell e.s.d.'s are taken into account individually in the estimation of e.s.d.'s in distances, angles and torsion angles; correlations between e.s.d.'s in cell parameters are only used when they are defined by crystal symmetry. An approximate (isotropic) treatment of cell e.s.d.'s is used for estimating e.s.d.'s involving l.s. planes.
Refinement. Refinement of *F*^2^ against ALL reflections. The weighted *R*-factor *wR* and goodness of fit *S* are based on *F*^2^, conventional *R*-factors *R* are based on *F*, with *F* set to zero for negative *F*^2^. The threshold expression of *F*^2^ > σ(*F*^2^) is used only for calculating *R*-factors(gt) *etc*. and is not relevant to the choice of reflections for refinement. *R*-factors based on *F*^2^ are statistically about twice as large as those based on *F*, and *R*- factors based on ALL data will be even larger.

### Fractional atomic coordinates and isotropic or equivalent isotropic displacement parameters (Å^2^)

**Table:**

	*x*	*y*	*z*	*U*_iso_*/*U*_eq_	Occ. (<1)
Mn	0.138711 (10)	0.44260 (4)	0.25751 (2)	0.03510 (14)	
S1	0.077589 (19)	0.17856 (7)	0.36556 (4)	0.0411 (2)	
N1	0.09707 (6)	0.5538 (2)	0.17267 (14)	0.0435 (6)	
N2	−0.05514 (7)	0.7148 (3)	−0.09856 (16)	0.0534 (7)	
N3	0.18000 (6)	0.3382 (2)	0.34637 (13)	0.0385 (6)	
N4	0.13043 (7)	0.9442 (3)	0.27057 (15)	0.0543 (7)	
H4N	0.1125	0.9505	0.2243	0.081*	
N5	0.27811 (7)	0.8985 (3)	0.57216 (17)	0.0593 (8)	
N6	0.07271 (6)	0.9608 (3)	0.15572 (15)	0.0510 (7)	
O1	0.16744 (5)	0.60471 (19)	0.29572 (11)	0.0489 (5)	
H1C	0.1850	0.6081	0.3420	0.073*	
H1D	0.1735	0.6465	0.2610	0.073*	
O2	0.15987 (5)	0.41069 (19)	0.17792 (11)	0.0442 (5)	
H2C	0.1660	0.4671	0.1603	0.066*	
H2D	0.1584	0.3426	0.1524	0.066*	
O3	0.11111 (5)	0.27534 (19)	0.22073 (11)	0.0457 (5)	
H3C	0.0932	0.2677	0.1802	0.069*	
H3D	0.1041	0.2381	0.2557	0.069*	
O4	0.11748 (5)	0.4668 (2)	0.33971 (11)	0.0485 (5)	
H4C	0.1000	0.4218	0.3420	0.073*	
H4D	0.1242	0.5011	0.3831	0.073*	
O5	0.16741 (6)	0.38646 (19)	0.60499 (12)	0.0535 (6)	
O6	0.17704 (6)	0.2475 (2)	0.69415 (12)	0.0604 (6)	
O7	0.12394 (6)	−0.2362 (2)	0.50527 (14)	0.0624 (7)	
O8	0.15990 (6)	−0.1909 (2)	0.61660 (14)	0.0616 (7)	
O9	0.04841 (6)	0.1151 (2)	0.35750 (13)	0.0613 (7)	
O10	0.09064 (6)	0.1395 (2)	0.31214 (12)	0.0592 (6)	
O11	0.07449 (6)	0.3107 (2)	0.36789 (13)	0.0566 (6)	
O1W	0.14542 (7)	0.5424 (2)	0.48152 (13)	0.0640 (7)	
H1A	0.1522	0.4881	0.5219	0.096*	
H1B	0.1400	0.6198	0.4980	0.096*	
O2W	0.07443 (8)	−0.2903 (3)	0.36216 (18)	0.0979 (10)	
H2A	0.0661	−0.3634	0.3735	0.147*	
H2B	0.0898	−0.2683	0.4068	0.147*	
O3W	0.02684 (10)	−0.1253 (3)	0.3116 (2)	0.1214 (13)	
H3A	0.0437	−0.1809	0.3167	0.182*	
H3B	0.0338	−0.0454	0.3323	0.182*	
O4W	0.02979 (10)	0.5171 (3)	0.3142 (3)	0.1261 (14)	
H4A	0.0372	0.4416	0.3155	0.189*	
H4B1	0.0090	0.5344	0.2805	0.189*	0.50
H4B2	0.0307	0.5587	0.2779	0.189*	0.50
O5W	0.22098 (10)	−0.1454 (6)	0.7192 (3)	0.184 (3)	
H5A	0.2045	−0.1664	0.6739	0.276*	
H5B	0.2303	−0.2172	0.7111	0.276*	
O6W	0.23851 (10)	0.1545 (6)	0.7469 (3)	0.176 (2)	
H6A	0.2159	0.1764	0.7353	0.264*	
H6B	0.2445	0.1076	0.7903	0.264*	
O7W	0.2369 (2)	0.4673 (9)	0.7033 (5)	0.304 (5)	
H7A	0.2183	0.4341	0.6714	0.456*	
H7B	0.2418	0.4293	0.7472	0.456*	
O8W	0.0000	0.2607 (10)	0.2500	0.248 (5)	
H8A	0.0160	0.2064	0.2737	0.371*	
C1	0.07441 (9)	0.6011 (4)	0.19136 (18)	0.0611 (10)	
H1	0.0783	0.6077	0.2413	0.073*	
C2	0.04520 (9)	0.6413 (4)	0.14079 (18)	0.0620 (10)	
H2	0.0302	0.6736	0.1574	0.074*	
C3	0.03820 (7)	0.6338 (3)	0.06573 (16)	0.0399 (7)	
C4	0.06260 (8)	0.5909 (3)	0.04616 (18)	0.0530 (9)	
H4	0.0600	0.5881	−0.0032	0.064*	
C5	0.09091 (8)	0.5522 (3)	0.10067 (18)	0.0543 (9)	
H5	0.1068	0.5230	0.0860	0.065*	
C6	−0.03092 (9)	0.6860 (4)	−0.11693 (19)	0.0635 (10)	
H6	−0.0345	0.6819	−0.1668	0.076*	
C7	−0.00038 (9)	0.6614 (4)	−0.06589 (19)	0.0623 (10)	
H7	0.0158	0.6422	−0.0820	0.075*	
C8	0.00604 (7)	0.6654 (3)	0.00895 (16)	0.0402 (7)	
C9	−0.01918 (8)	0.6976 (3)	0.02817 (18)	0.0525 (8)	
H9	−0.0163	0.7032	0.0776	0.063*	
C10	−0.04883 (8)	0.7212 (4)	−0.0266 (2)	0.0589 (9)	
H10	−0.0654	0.7430	−0.0122	0.071*	
C11	0.20872 (7)	0.3379 (3)	0.34306 (16)	0.0423 (7)	
H11	0.2103	0.3628	0.2993	0.051*	
C12	0.23632 (7)	0.3028 (3)	0.40062 (16)	0.0425 (7)	
H12	0.2557	0.3038	0.3947	0.051*	
C13	0.23533 (7)	0.2660 (3)	0.46751 (15)	0.0350 (6)	
C14	0.20507 (7)	0.2620 (3)	0.47004 (17)	0.0495 (8)	
H14	0.2027	0.2352	0.5126	0.059*	
C15	0.17869 (7)	0.2977 (3)	0.40957 (17)	0.0477 (8)	
H15	0.1588	0.2935	0.4128	0.057*	
C16	0.13366 (8)	0.8674 (4)	0.3250 (2)	0.0559 (9)	
H16	0.1164	0.8195	0.3230	0.067*	
C17	0.16217 (8)	0.8577 (3)	0.38458 (18)	0.0500 (8)	
H17	0.1642	0.8032	0.4224	0.060*	
C18	0.18801 (7)	0.9292 (3)	0.38830 (16)	0.0406 (7)	
C19	0.18382 (8)	1.0080 (3)	0.32978 (17)	0.0478 (8)	
H19	0.2008	1.0560	0.3301	0.057*	
C20	0.15476 (8)	1.0156 (3)	0.27148 (18)	0.0531 (8)	
H20	0.1519	1.0696	0.2329	0.064*	
C21	0.27635 (8)	0.9214 (3)	0.5038 (2)	0.0579 (9)	
H21	0.2953	0.9296	0.4964	0.069*	
C22	0.24774 (8)	0.9335 (3)	0.44329 (19)	0.0513 (8)	
H22	0.2478	0.9501	0.3969	0.062*	
C23	0.21924 (7)	0.9207 (3)	0.45215 (17)	0.0420 (7)	
C24	0.22095 (9)	0.8982 (3)	0.52304 (18)	0.0562 (9)	
H24	0.2024	0.8897	0.5321	0.067*	
C25	0.25060 (10)	0.8885 (4)	0.5803 (2)	0.0650 (10)	
H25	0.2512	0.8740	0.6275	0.078*	
C26	0.07059 (8)	0.9386 (3)	0.08743 (18)	0.0535 (9)	
H26	0.0888	0.9120	0.0811	0.064*	
C27	0.04305 (8)	0.9528 (3)	0.02553 (18)	0.0511 (8)	
H27	0.0431	0.9365	−0.0209	0.061*	
C28	0.01528 (7)	0.9915 (3)	0.03266 (15)	0.0375 (6)	
C29	0.01739 (8)	1.0117 (4)	0.10414 (18)	0.0573 (9)	
H29	−0.0006	1.0358	0.1123	0.069*	
C30	0.04590 (8)	0.9965 (4)	0.16272 (19)	0.0641 (10)	
H30	0.0466	1.0118	0.2099	0.077*	
C31	0.10688 (7)	0.1346 (3)	0.45289 (15)	0.0353 (6)	
C32	0.12452 (7)	0.2216 (2)	0.50320 (15)	0.0357 (6)	
H32	0.1210	0.3042	0.4919	0.043*	
C33	0.14755 (7)	0.1851 (2)	0.57097 (15)	0.0351 (6)	
C34	0.15267 (7)	0.0610 (3)	0.58662 (16)	0.0370 (6)	
H34	0.1680	0.0365	0.6317	0.044*	
C35	0.13527 (7)	−0.0271 (2)	0.53592 (15)	0.0350 (6)	
C36	0.11214 (7)	0.0109 (3)	0.46826 (15)	0.0377 (6)	
H36	0.1003	−0.0467	0.4337	0.045*	
C37	0.16561 (7)	0.2811 (3)	0.62790 (17)	0.0413 (7)	
C38	0.14015 (8)	−0.1624 (3)	0.55409 (17)	0.0418 (7)	

### Atomic displacement parameters (Å^2^)

**Table:**

	*U*^11^	*U*^22^	*U*^33^	*U*^12^	*U*^13^	*U*^23^
Mn	0.0311 (3)	0.0407 (3)	0.0270 (2)	0.00423 (18)	0.00411 (19)	0.00406 (17)
S1	0.0453 (5)	0.0417 (4)	0.0313 (4)	−0.0020 (3)	0.0092 (3)	0.0049 (3)
N1	0.0369 (14)	0.0462 (15)	0.0363 (14)	0.0040 (11)	0.0020 (11)	0.0056 (11)
N2	0.0395 (16)	0.0569 (17)	0.0469 (17)	−0.0001 (13)	−0.0020 (13)	0.0070 (13)
N3	0.0332 (13)	0.0431 (14)	0.0318 (13)	0.0051 (10)	0.0042 (11)	0.0060 (10)
N4	0.0392 (16)	0.071 (2)	0.0411 (16)	0.0109 (14)	0.0028 (13)	−0.0122 (14)
N5	0.0526 (19)	0.0516 (17)	0.0507 (18)	0.0002 (14)	−0.0053 (15)	−0.0014 (14)
N6	0.0371 (15)	0.0639 (18)	0.0423 (15)	−0.0018 (13)	0.0047 (13)	−0.0041 (13)
O1	0.0510 (13)	0.0471 (12)	0.0346 (11)	−0.0066 (10)	0.0011 (10)	0.0019 (9)
O2	0.0596 (14)	0.0369 (11)	0.0387 (11)	0.0034 (10)	0.0218 (10)	0.0017 (9)
O3	0.0364 (12)	0.0540 (13)	0.0362 (11)	−0.0062 (9)	0.0025 (9)	0.0061 (9)
O4	0.0464 (13)	0.0640 (14)	0.0360 (11)	0.0061 (11)	0.0170 (10)	0.0026 (10)
O5	0.0730 (16)	0.0342 (12)	0.0452 (13)	−0.0066 (11)	0.0140 (12)	−0.0056 (10)
O6	0.0799 (18)	0.0473 (13)	0.0360 (13)	0.0062 (12)	0.0025 (12)	−0.0052 (10)
O7	0.0805 (18)	0.0325 (12)	0.0585 (15)	−0.0010 (11)	0.0095 (13)	−0.0051 (11)
O8	0.0707 (17)	0.0367 (12)	0.0575 (15)	0.0002 (11)	0.0028 (13)	0.0124 (11)
O9	0.0461 (14)	0.0736 (17)	0.0518 (14)	−0.0143 (12)	0.0051 (11)	0.0143 (12)
O10	0.0767 (17)	0.0676 (16)	0.0357 (12)	0.0012 (13)	0.0242 (12)	0.0035 (11)
O11	0.0618 (15)	0.0436 (13)	0.0531 (14)	0.0059 (11)	0.0098 (12)	0.0089 (10)
O1W	0.093 (2)	0.0526 (14)	0.0434 (13)	0.0145 (13)	0.0225 (13)	−0.0021 (11)
O2W	0.099 (2)	0.102 (2)	0.081 (2)	0.014 (2)	0.0209 (19)	−0.0076 (18)
O3W	0.120 (3)	0.082 (2)	0.138 (3)	0.006 (2)	0.024 (3)	−0.013 (2)
O4W	0.127 (3)	0.082 (2)	0.181 (4)	0.018 (2)	0.073 (3)	0.013 (3)
O5W	0.078 (3)	0.310 (8)	0.134 (4)	−0.028 (4)	0.007 (3)	0.086 (5)
O6W	0.075 (3)	0.310 (8)	0.136 (4)	0.028 (4)	0.032 (3)	0.044 (4)
O7W	0.242 (8)	0.326 (11)	0.304 (10)	−0.131 (8)	0.061 (8)	0.011 (9)
O8W	0.200 (9)	0.213 (10)	0.249 (12)	0.000	−0.003 (8)	0.000
C1	0.060 (2)	0.078 (3)	0.0308 (17)	0.0277 (19)	0.0012 (16)	−0.0017 (16)
C2	0.051 (2)	0.085 (3)	0.0391 (18)	0.0325 (19)	0.0055 (16)	−0.0021 (17)
C3	0.0355 (16)	0.0371 (16)	0.0384 (16)	0.0034 (12)	0.0046 (13)	0.0047 (12)
C4	0.0399 (19)	0.077 (2)	0.0371 (17)	0.0061 (16)	0.0096 (15)	0.0162 (16)
C5	0.0356 (18)	0.081 (3)	0.0416 (18)	0.0125 (16)	0.0102 (15)	0.0181 (17)
C6	0.054 (2)	0.085 (3)	0.0347 (18)	0.010 (2)	−0.0013 (16)	0.0057 (17)
C7	0.049 (2)	0.087 (3)	0.0408 (19)	0.0163 (19)	0.0059 (16)	0.0024 (18)
C8	0.0339 (16)	0.0402 (16)	0.0353 (16)	0.0029 (12)	0.0010 (13)	0.0039 (12)
C9	0.0409 (19)	0.069 (2)	0.0393 (18)	0.0041 (16)	0.0064 (15)	0.0067 (16)
C10	0.0368 (19)	0.077 (3)	0.055 (2)	0.0055 (17)	0.0086 (16)	0.0080 (18)
C11	0.0365 (17)	0.0562 (19)	0.0313 (15)	0.0081 (14)	0.0096 (13)	0.0088 (13)
C12	0.0342 (16)	0.0576 (19)	0.0346 (15)	0.0096 (14)	0.0118 (13)	0.0078 (13)
C13	0.0338 (15)	0.0343 (14)	0.0324 (14)	0.0055 (12)	0.0077 (12)	0.0052 (11)
C14	0.0361 (17)	0.071 (2)	0.0382 (17)	0.0099 (15)	0.0106 (14)	0.0234 (15)
C15	0.0283 (16)	0.067 (2)	0.0424 (17)	0.0057 (14)	0.0074 (14)	0.0182 (15)
C16	0.0390 (19)	0.070 (2)	0.052 (2)	−0.0057 (16)	0.0105 (16)	−0.0099 (18)
C17	0.0428 (19)	0.058 (2)	0.0432 (18)	−0.0046 (15)	0.0096 (15)	−0.0019 (15)
C18	0.0375 (17)	0.0429 (17)	0.0349 (16)	0.0026 (13)	0.0066 (13)	−0.0059 (12)
C19	0.0437 (19)	0.0511 (19)	0.0413 (17)	0.0028 (15)	0.0082 (15)	−0.0007 (14)
C20	0.051 (2)	0.060 (2)	0.0385 (18)	0.0099 (17)	0.0057 (16)	−0.0009 (15)
C21	0.0384 (19)	0.062 (2)	0.060 (2)	0.0014 (16)	0.0045 (17)	−0.0070 (17)
C22	0.0433 (19)	0.059 (2)	0.0431 (18)	0.0006 (15)	0.0078 (16)	−0.0035 (15)
C23	0.0374 (17)	0.0406 (16)	0.0375 (16)	0.0017 (13)	0.0029 (14)	−0.0038 (13)
C24	0.052 (2)	0.067 (2)	0.0419 (19)	−0.0088 (17)	0.0095 (16)	0.0017 (16)
C25	0.063 (3)	0.075 (3)	0.0391 (19)	−0.009 (2)	0.0002 (18)	0.0062 (17)
C26	0.0316 (17)	0.079 (2)	0.0457 (19)	0.0073 (16)	0.0097 (15)	−0.0011 (17)
C27	0.0361 (18)	0.076 (2)	0.0389 (17)	0.0055 (16)	0.0121 (15)	−0.0056 (16)
C28	0.0312 (15)	0.0409 (16)	0.0368 (15)	−0.0032 (12)	0.0094 (12)	−0.0058 (12)
C29	0.0354 (18)	0.094 (3)	0.0387 (18)	0.0029 (17)	0.0100 (15)	−0.0117 (18)
C30	0.041 (2)	0.109 (3)	0.0362 (18)	0.004 (2)	0.0088 (16)	−0.0102 (19)
C31	0.0356 (16)	0.0376 (15)	0.0325 (15)	−0.0010 (12)	0.0131 (13)	0.0007 (12)
C32	0.0435 (17)	0.0283 (14)	0.0356 (15)	0.0007 (12)	0.0154 (13)	0.0004 (11)
C33	0.0378 (16)	0.0342 (15)	0.0324 (14)	−0.0007 (12)	0.0127 (13)	−0.0017 (11)
C34	0.0385 (16)	0.0365 (15)	0.0335 (15)	0.0016 (12)	0.0112 (13)	0.0020 (12)
C35	0.0376 (16)	0.0293 (14)	0.0372 (15)	0.0003 (12)	0.0134 (13)	0.0007 (12)
C36	0.0441 (17)	0.0323 (15)	0.0347 (15)	−0.0042 (12)	0.0131 (13)	−0.0046 (12)
C37	0.0424 (18)	0.0372 (17)	0.0394 (17)	0.0018 (13)	0.0102 (14)	−0.0057 (13)
C38	0.0455 (18)	0.0336 (16)	0.0468 (18)	−0.0014 (13)	0.0183 (16)	0.0006 (13)

### Geometric parameters (Å, °)

**Table:**

Mn—N1	2.323 (2)	C4—C5	1.384 (4)
Mn—N3	2.311 (2)	C4—H4	0.9300
Mn—O1	2.158 (2)	C5—H5	0.9300
Mn—O2	2.156 (2)	C6—C7	1.388 (5)
Mn—O3	2.178 (2)	C6—H6	0.9300
Mn—O4	2.192 (2)	C7—C8	1.384 (4)
S1—O9	1.449 (2)	C7—H7	0.9300
S1—O10	1.454 (2)	C8—C9	1.382 (4)
S1—O11	1.455 (2)	C9—C10	1.385 (5)
S1—C31	1.784 (3)	C9—H9	0.9300
N1—C1	1.324 (4)	C10—H10	0.9300
N1—C5	1.331 (4)	C11—C12	1.379 (4)
N2—C6	1.321 (5)	C11—H11	0.9300
N2—C10	1.331 (5)	C12—C13	1.391 (4)
N3—C11	1.330 (4)	C12—H12	0.9300
N3—C15	1.341 (4)	C13—C14	1.395 (4)
N4—C16	1.323 (5)	C13—C13^i^	1.487 (5)
N4—C20	1.348 (5)	C14—C15	1.378 (4)
N4—H4N	0.9591	C14—H14	0.9300
N5—C25	1.323 (5)	C15—H15	0.9300
N5—C21	1.338 (5)	C16—C17	1.373 (5)
N6—C26	1.329 (4)	C16—H16	0.9300
N6—C30	1.334 (4)	C17—C18	1.388 (4)
O1—H1C	0.9498	C17—H17	0.9300
O1—H1D	0.9449	C18—C19	1.391 (4)
O2—H2C	0.8083	C18—C23	1.489 (4)
O2—H2D	0.8864	C19—C20	1.376 (5)
O3—H3C	0.8966	C19—H19	0.9300
O3—H3D	0.9540	C20—H20	0.9300
O4—H4C	0.9492	C21—C22	1.387 (5)
O4—H4D	0.8713	C21—H21	0.9300
O5—C37	1.253 (4)	C22—C23	1.378 (5)
O6—C37	1.256 (4)	C22—H22	0.9300
O7—C38	1.253 (4)	C23—C24	1.386 (5)
O8—C38	1.248 (4)	C24—C25	1.385 (5)
O1W—H1A	0.9430	C24—H24	0.9300
O1W—H1B	0.9721	C25—H25	0.9300
O2W—H2A	0.9482	C26—C27	1.375 (5)
O2W—H2B	0.9176	C26—H26	0.9300
O3W—H3A	0.9515	C27—C28	1.387 (4)
O3W—H3B	0.9647	C27—H27	0.9300
O4W—H4A	0.8889	C28—C29	1.388 (4)
O4W—H4B1	0.9379	C28—C28^ii^	1.494 (6)
O4W—H4B2	0.8595	C29—C30	1.370 (5)
O5W—H5A	0.9434	C29—H29	0.9300
O5W—H5B	0.9357	C30—H30	0.9300
O6W—H6A	0.9924	C31—C32	1.383 (4)
O6W—H6B	0.9409	C31—C36	1.388 (4)
O7W—H7A	0.9119	C32—C33	1.396 (4)
O7W—H7B	0.9034	C32—H32	0.9300
O8W—H8A	0.9147	C33—C34	1.392 (4)
C1—C2	1.387 (5)	C33—C37	1.522 (4)
C1—H1	0.9300	C34—C35	1.392 (4)
C2—C3	1.385 (4)	C34—H34	0.9300
C2—H2	0.9300	C35—C36	1.402 (4)
C3—C4	1.387 (4)	C35—C38	1.520 (4)
C3—C8	1.496 (4)	C36—H36	0.9300
			
O2—Mn—O1	90.39 (8)	N3—C11—C12	123.9 (3)
O2—Mn—O3	89.06 (8)	N3—C11—H11	118.0
O1—Mn—O3	178.12 (9)	C12—C11—H11	118.0
O2—Mn—O4	177.57 (8)	C11—C12—C13	120.3 (3)
O1—Mn—O4	91.31 (9)	C11—C12—H12	119.8
O3—Mn—O4	89.19 (9)	C13—C12—H12	119.8
O2—Mn—N3	90.52 (8)	C12—C13—C14	115.7 (3)
O1—Mn—N3	86.50 (9)	C12—C13—C13^i^	122.3 (3)
O3—Mn—N3	91.70 (8)	C14—C13—C13^i^	122.0 (3)
O4—Mn—N3	87.85 (8)	C15—C14—C13	120.0 (3)
O2—Mn—N1	91.81 (9)	C15—C14—H14	120.0
O1—Mn—N1	91.94 (9)	C13—C14—H14	120.0
O3—Mn—N1	89.87 (9)	N3—C15—C14	123.9 (3)
O4—Mn—N1	89.87 (9)	N3—C15—H15	118.1
N3—Mn—N1	177.21 (9)	C14—C15—H15	118.1
O9—S1—O10	112.96 (16)	N4—C16—C17	120.8 (3)
O9—S1—O11	112.59 (16)	N4—C16—H16	119.6
O10—S1—O11	112.72 (14)	C17—C16—H16	119.6
O9—S1—C31	106.63 (13)	C16—C17—C18	120.1 (3)
O10—S1—C31	104.74 (14)	C16—C17—H17	120.0
O11—S1—C31	106.46 (14)	C18—C17—H17	120.0
C1—N1—C5	115.6 (3)	C17—C18—C19	117.6 (3)
C1—N1—Mn	120.7 (2)	C17—C18—C23	121.3 (3)
C5—N1—Mn	122.3 (2)	C19—C18—C23	121.1 (3)
C6—N2—C10	116.2 (3)	C20—C19—C18	120.4 (3)
C11—N3—C15	116.0 (2)	C20—C19—H19	119.8
C11—N3—Mn	119.27 (18)	C18—C19—H19	119.8
C15—N3—Mn	123.37 (19)	N4—C20—C19	119.6 (3)
C16—N4—C20	121.5 (3)	N4—C20—H20	120.2
C16—N4—H4N	126.9	C19—C20—H20	120.2
C20—N4—H4N	111.2	N5—C21—C22	123.4 (4)
C25—N5—C21	116.3 (3)	N5—C21—H21	118.3
C26—N6—C30	116.3 (3)	C22—C21—H21	118.3
Mn—O1—H1C	122.4	C23—C22—C21	119.8 (3)
Mn—O1—H1D	116.8	C23—C22—H22	120.1
H1C—O1—H1D	107.6	C21—C22—H22	120.1
Mn—O2—H2C	120.7	C22—C23—C24	117.0 (3)
Mn—O2—H2D	126.1	C22—C23—C18	121.5 (3)
H2C—O2—H2D	111.3	C24—C23—C18	121.5 (3)
Mn—O3—H3C	125.6	C25—C24—C23	119.3 (3)
Mn—O3—H3D	115.5	C25—C24—H24	120.4
H3C—O3—H3D	99.0	C23—C24—H24	120.4
Mn—O4—H4C	125.8	N5—C25—C24	124.2 (3)
Mn—O4—H4D	133.2	N5—C25—H25	117.9
H4C—O4—H4D	99.3	C24—C25—H25	117.9
H1A—O1W—H1B	108.1	N6—C26—C27	123.9 (3)
H2A—O2W—H2B	102.8	N6—C26—H26	118.0
H3A—O3W—H3B	114.7	C27—C26—H26	118.0
H4A—O4W—H4B1	118.4	C26—C27—C28	119.8 (3)
H4A—O4W—H4B2	112.1	C26—C27—H27	120.1
H4B1—O4W—H4B2	70.4	C28—C27—H27	120.1
H5A—O5W—H5B	83.2	C27—C28—C29	116.1 (3)
H6A—O6W—H6B	104.5	C27—C28—C28^ii^	122.0 (3)
H7A—O7W—H7B	107.1	C29—C28—C28^ii^	121.8 (3)
H8A—O8W—H8A^iii^	98.91	C30—C29—C28	120.3 (3)
N1—C1—C2	123.8 (3)	C30—C29—H29	119.9
N1—C1—H1	118.1	C28—C29—H29	119.9
C2—C1—H1	118.1	N6—C30—C29	123.5 (3)
C3—C2—C1	120.4 (3)	N6—C30—H30	118.2
C3—C2—H2	119.8	C29—C30—H30	118.2
C1—C2—H2	119.8	C32—C31—C36	120.8 (3)
C2—C3—C4	115.8 (3)	C32—C31—S1	120.8 (2)
C2—C3—C8	122.4 (3)	C36—C31—S1	118.3 (2)
C4—C3—C8	121.7 (3)	C31—C32—C33	119.9 (3)
C5—C4—C3	119.4 (3)	C31—C32—H32	120.1
C5—C4—H4	120.3	C33—C32—H32	120.1
C3—C4—H4	120.3	C34—C33—C32	119.3 (3)
N1—C5—C4	124.7 (3)	C34—C33—C37	121.0 (3)
N1—C5—H5	117.7	C32—C33—C37	119.7 (3)
C4—C5—H5	117.7	C35—C34—C33	121.2 (3)
N2—C6—C7	123.6 (3)	C35—C34—H34	119.4
N2—C6—H6	118.2	C33—C34—H34	119.4
C7—C6—H6	118.2	C34—C35—C36	118.9 (3)
C8—C7—C6	120.2 (3)	C34—C35—C38	121.1 (3)
C8—C7—H7	119.9	C36—C35—C38	120.0 (3)
C6—C7—H7	119.9	C31—C36—C35	119.9 (3)
C9—C8—C7	116.2 (3)	C31—C36—H36	120.0
C9—C8—C3	121.9 (3)	C35—C36—H36	120.0
C7—C8—C3	121.9 (3)	O5—C37—O6	125.7 (3)
C8—C9—C10	119.6 (3)	O5—C37—C33	117.6 (3)
C8—C9—H9	120.2	O6—C37—C33	116.7 (3)
C10—C9—H9	120.2	O8—C38—O7	125.4 (3)
N2—C10—C9	124.1 (3)	O8—C38—C35	117.3 (3)
N2—C10—H10	117.9	O7—C38—C35	117.4 (3)
C9—C10—H10	117.9		

Symmetry codes: (i) −*x*+1/2, −*y*+1/2, −*z*+1; (ii) −*x*, −*y*+2, −*z*; (iii) −*x*, *y*, −*z*+1/2.

### Hydrogen-bond geometry (Å, °)

**Table:**

*D*—H···*A*	*D*—H	H···*A*	*D*···*A*	*D*—H···*A*
N4—H4N···N6	0.96	1.79	2.725 (4)	163
O1—H1C···N5^iv^	0.95	1.86	2.809 (4)	173
O1—H1D···O6^v^	0.95	1.81	2.731 (3)	165
O2—H2C···O5^v^	0.81	1.95	2.735 (3)	163
O2—H2D···O8^vi^	0.89	1.81	2.691 (3)	169
O3—H3C···N2^vii^	0.90	1.86	2.742 (4)	168
O3—H3D···O10	0.95	1.81	2.754 (3)	169
O4—H4C···O11	0.95	1.88	2.805 (4)	164
O4—H4D···O1W	0.87	1.85	2.706 (3)	166
O1W—H1A···O5	0.94	1.87	2.815 (3)	177
O1W—H1B···O7^viii^	0.97	1.77	2.719 (3)	166
O2W—H2A···O4W^ix^	0.95	2.07	2.822 (6)	135
O2W—H2B···O7	0.92	1.99	2.903 (4)	175
O3W—H3A···O2W	0.95	1.79	2.694 (5)	157
O3W—H3B···O9	0.96	1.88	2.831 (4)	170
O4W—H4A···O11	0.89	2.15	2.946 (5)	148
O4W—H4B1···O4W^x^	0.94	2.02	2.900 (8)	156
O5W—H5A···O8	0.94	1.92	2.772 (6)	149
O5W—H5B···O6W^xi^	0.93	1.94	2.771 (9)	147
O6W—H6A···O6	0.99	1.81	2.768 (6)	162
O6W—H6B···O7W^xi^	0.94	1.73	2.358 (12)	121
O7W—H7A···O5	0.91	2.23	3.124 (10)	166
O7W—H7B···O5W^xii^	0.90	1.76	2.291 (11)	115
O8W—H8A···O9	0.91	2.00	2.871 (7)	159

Symmetry codes: (iv) −*x*+1/2, −*y*+3/2, −*z*+1; (v) *x*, −*y*+1, *z*−1/2; (vi) *x*, −*y*, *z*−1/2; (vii) −*x*, −*y*+1, −*z*; (viii) *x*, *y*+1, *z*; (ix) *x*, *y*−1, *z*; (x) −*x*, *y*, −*z*+1/2; (xi) −*x*+1/2, *y*−1/2, −*z*+3/2; (xii) −*x*+1/2, *y*+1/2, −*z*+3/2.

### Table 3 Table 3. A summar of the distances and angles between partially overlapped pyridine rings (Å, °)

**Table:**

Ring (I)	Ring (J)	Angle	Perp (I)	Perp (J)	Cg–Cg
N1-pyridine	N2^i^-pyridine	8.29	3.404	3.491	3.691 (2)
N2-pyridine	N6^ii^-pyridine	5.33	3.403	3.391	3.794 (2)
N3-pyridine	N5^iii^-pyridine	10.91	3.260	3.477	3.751 (2)
N5-pyridine	N5^i^-pyridine	0.00	3.544	3.544	3.547 (2)

Symmetry codes: (i) -x, 1-y, -z; (ii) -x, 2-y, -z; (iii) 1/2-x, 3/2-y, 1-z. Notes: Angle: dihedral angle between ring (I) and ring (J). Perp(I) is the perpendicular distance of centroid of ring (I) on ring (J). Perp(J) is the perpendicular distance of centroid of ring (J) on ring (I). Cg–Cg is the distance between centroids of ring (I) and ring (J).
